# The maintenance of genetic polymorphism underlying sexually antagonistic traits

**DOI:** 10.1093/evlett/qrae059

**Published:** 2024-12-18

**Authors:** Ewan Flintham, Vincent Savolainen, Sarah P Otto, Max Reuter, Charles Mullon

**Affiliations:** Department of Ecology and Evolution, University of Lausanne, Lausanne, Switzerland; Department of Life Sciences, Georgina Mace Centre for the Living Planet, Silwood Park Campus, Imperial College London, Ascot, United Kingdom; Department of Life Sciences, Georgina Mace Centre for the Living Planet, Silwood Park Campus, Imperial College London, Ascot, United Kingdom; Department of Zoology, and Biodiversity Research Centre, University of British Columbia, Vancouver, British Columbia, Canada; Research Department of Genetics, Evolution and Environment, University College London, London, United Kingdom; Department of Ecology and Evolution, University of Lausanne, Lausanne, Switzerland

**Keywords:** polymorphism, genetic variation, balancing selection, sexual conflict, negative frequency dependence, sexual dimorphism

## Abstract

Selection often favors different trait values in males and females, leading to genetic conflicts between the sexes when traits have a shared genetic basis. Such sexual antagonism has been proposed to maintain genetic polymorphism. However, this notion is based on insights from population genetic models of single loci with fixed fitness effects. It is thus unclear how readily polymorphism emerges from sex-specific selection acting on continuous traits, where fitness effects arise from the genotype-phenotype map and the fitness landscape. Here, we model the evolution of a continuous trait that has a shared genetic basis but different optima in males and females, considering a wide variety of genetic architectures and fitness landscapes. For autosomal loci, the long-term maintenance of polymorphism requires strong conflict between males and females that generates uncharacteristic sex-specific fitness patterns. Instead, more plausible sex-specific fitness landscapes typically generate stabilizing selection leading to an evolutionarily stable state that consists of a single homozygous genotype. Except for sites tightly linked to the sex-determining region, our results indicate that genetic variation due to sexual antagonism should arise only rarely and often be transient, making these signatures challenging to detect in genomic data.

## Introduction

In sexual populations, genetic conflict can arise between males and females when traits that are genetically correlated across the sexes show different optima (Bonduriansky & Chenoweth, 2009; Cox & Calsbeek, 2009; Poissant et al., 2010). Here, alleles with similar phenotypic effects in males and females can be favored in one sex but disfavored in the other (Arnqvist & Rowe, 2005; Bonduriansky & Chenoweth, 2009; Chapman et al., 2003; Connallon & Clark, 2014a; Parker et al., 1979; Rice & Chippindale, 2001; Van Doorn, 2009). Such “sexual antagonism” (Connallon & Clark, 2014a,b; Muralidhar & Coop, 2024) is thought to have wide-ranging ecological and evolutionary implications, from driving a fitness load across the sexes (Bonduriansky & Chenoweth, 2009; Lande, 1980) to shaping patterns of genetic variation within, and even between, species (Bonduriansky & Chenoweth, 2009; Connallon & Clark, 2014b; Mank, 2017a, see Eyer et al., 2019; Foerster et al., 2007; Ruzicka et al., 2019 for empirical examples).

The potential influence of sexually antagonistic selection on genetic variation has been highlighted by multiple population genetic models (Albert & Otto, 2005; Arnqvist et al., 2014; Connallon & Clark, 2012; Flintham et al., 2021; Fry, 2010; Gavrilets & Rice, 2006; Hill et al., 2019; Hitchcock & Gardner, 2020; Jaquiéry et al., 2013; Jordan & Connallon, 2014; Kidwell et al., 1977; Mullon et al., 2012; Otto, 2019; Owen, 1953; Patten & Haig, 2009; Patten et al., 2010; Rice, 1984; Ruzicka & Connallon, 2020; Spencer & Priest, 2016; Ubeda et al., 2011). One salient point from this theory is that sexual antagonism can maintain a balanced genetic polymorphism for two alleles at a locus either when selection is strong and symmetrical between the sexes or when allelic dominance is sex-specific such that alleles are more dominant when beneficial and more recessive when deleterious (“sex-specific dominance reversal” Arnqvist et al., 2014; Connallon & Chenoweth, 2019; Fry, 2010; Kidwell et al., 1977). Models of trait evolution (Connallon & Clark, 2014b; Connallon & Chenoweth, 2019) have found that such dominance relationships frequently emerge during the course of sex-specific adaptation in the weak-mutation strong-selection limit (i.e., such that a maximum of one di-allelic locus segregates at any given time) if fitness landscapes decline slowly away from phenotypic optima (e.g., Gaussian fitness landscapes). This has led to the suggestion that sexually antagonistic balancing selection is a common outcome of sex-specific selection (Arnqvist et al., 2014; Connallon & Clark, 2014b; Connallon & Chenoweth, 2019; Grieshop & Arnqvist, 2018; Grieshop et al., 2024; Kaufmann et al., 2023; Mank, 2017a; Reid, 2022), including selection acting on traits influenced by multiple segregating loci or by loci at which alleles with a range of phenotypic effects may arise over time (Cox & Calsbeek, 2009; Khramtsova et al., 2019; Stearns et al., 2012, i.e., multi-allelic or continuous traits Charlesworth & Charlesworth, 2010; Walsh & Lynch, 2018).

In contrast to alleles at a single di-allelic locus, however, the fitness consequences of alleles underlying polygenic or continuous traits are not straightforward, being determined by the genotype-phenotype map and the male and female fitness landscapes (Walsh & Lynch, 2018). This calls into question whether insights into the maintenance of genetic variation from single-locus theory readily extend to traits with a more complex basis. For example, multi-locus population genetic modeling suggests that variation is reduced across loci when males and females have different genotype-phenotype maps but selection is stabilizing for the same trait value in both sexes (Turelli & Barton, 2004). Quantitative genetic models investigating sex-specific adaptation in complex traits, meanwhile, provide little information with regard to the maintenance of genetic variation as variation is assumed constant through time (i.e., that traits are determined by many loci of small effect such that cross-sex genetic correlations are constant Connallon & Matthews, 2019; Lande, 1980; Matthews et al., 2019). Bridging population and quantitative genetic models, recent simulation work has shown how sexual dimorphism in a polygenic trait can evolve outside of the weak-mutation strong-selection limit but does not consider the potential for unresolved sexual antagonism to maintain variation (Muralidhar & Coop, 2024).

Here, we study the maintenance of genetic variation for a continuous trait subject to male- and female-specific fitness landscapes with a shared genetic basis across the sexes, such that genetic conflict emerges. Using a combination of mathematical analyses and multi-locus simulations, we show that sexually antagonistic selection should rarely drive polymorphism when traits are polygenic or where loci undergo continual mutation. Rather, our results suggest that sexually antagonistic selection will typically diminish genetic variation unless traits are determined by very few loci experiencing strong genetic constraints (e.g., where very few alleles can arise through mutation). Our findings further imply that detecting sexually antagonistic variation from genomic data of natural populations will likely remain challenging because such polymorphism should be transient.

Box: 1. **Fecundity power functions**

**Fig 1:**
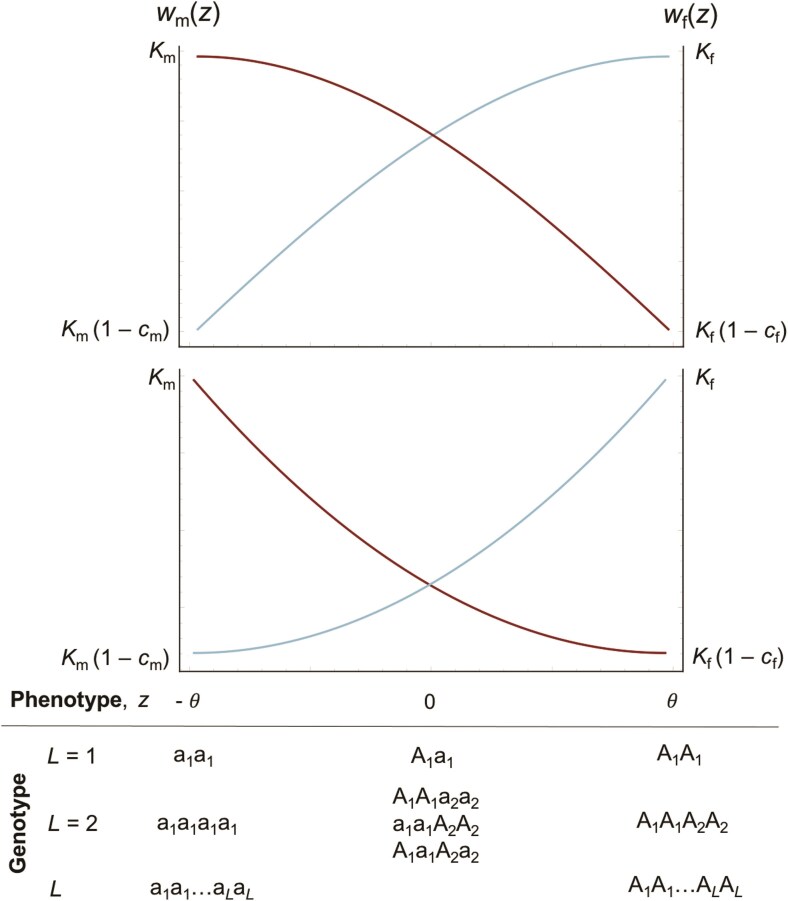
Sex-specific fecundity landscapes. Male (red curves) and female (blue curves) fecundity as a function of phenotype, z, Equation (I.A). A male’s fecundity is greatest when he expresses the phenotype z=-θ, that is, he is homozygous for allele “a” at every locus, while a female’s fecundity is maximized when she expresses z=θ, that is, she is homozygous for allele “A” at all loci (Equations (2) and (3)). Top and bottom panels show diminishing ($b_{m}=b_{f}<1$) and accelerating ($b_{m}=b_{f}>1$) returns to adaptation in each sex, respectively.

To illustrate how different fitness landscapes influence outcomes of sexual antagonism, we introduce the power functions

$$w_{m}(z)=K_{m}\left(1-c_{m}\left[1-{\left(\frac{\theta -z}{2\theta }\right)}^{b_{m}}\right]\right),w_{f}(z)=K_{f}\left(1-c_{f}\left[1-{\left(\frac{\theta +z}{2\theta }\right)}^{b_{f}}\right]\right),$$ (I.A)

where $K_{m}$ and $K_{f}$ are the maximum number of gametes produced by a male and a female (both assumed to be large). According to Equation (I.A), $w_{m}(z)$ decreases from $K_{m}$ to $(1-c_{m})K_{m}$ as z increases from -θ, whereas $w_{f}(z)$ decreases from $K_{f}$ to $K_{f}(1-c_{f})$ as z decreases from θ (Figure 1). Here, we assume that z is bounded to -θ≤z≤θ so that our analyses are focused on the trait space between the male and female optima where selection is sexually antagonistic. The coefficients $0\leq c_{m}\leq 1$ and $0\leq c_{f}\leq 1$ tune the steepness of the fitness decline in each sex and so the strength of the trade-off over male and female fecundity (see Fry, 2010; Kidwell et al., 1977 for a similar approach for a single locus). Finally, $b_{m}$ and $b_{f}$ control the shape of the sex-specific landscapes between θ and -θ, such that landscapes are accelerating in males and females when $b_{m}>1$ and $b_{f}>1$ and diminishing when $b_{m}<1$ and $b_{f}<1$. For example, $b_{f}<1$ and $b_{f}>1$ would correspond to negative and positive second-order effects in a quadratic regression of female fitness on the trait z (Lande & Arnold, 1983). To aid the presentation, we focus in the main text on the case where landscapes have the same shape in both sexes ($b_{m}=b_{f}=b$); this assumption is relaxed in Supplementary Appendix C.3.

## Model

### Life-cycle, traits, and sex-specific fitness landscape

We consider a diploid population of large and constant size that is composed of equal numbers of adult males and females. At each generation, adult males and females produce large numbers of gametes that fuse randomly to produce zygotes. These zygotes become male and female juveniles with equal probability (so that the sex ratio at birth is unbiased). Adults die and juveniles compete randomly within each sex to become the male and female adults of the next generation.

Each individual expresses a continuous trait, z∈ℝ, that influences their fitness. We consider a scenario whereby this trait affects the number of gametes produced (i.e., fecundity), but trait effects on fitness could equivalently be through survival and/or competition (e.g., for breeding spots) within sexes (as in Bodmer, 1965; Connallon & Clark, 2014b; Haldane, 1924; Kidwell et al., 1977; Muralidhar & Coop, 2024; Rice, 1984). Male and female fecundity may depend on trait z differently, and we denote the fecundity of a male with trait z as $w_{m}(z)$ and fecundity of a female with trait z as $w_{f}(z)$. The functions $w_{m}(z)$ and $w_{f}(z)$ thus characterize the male and female fitness landscape, respectively. We focus on a range of trait values over which there is a conflict between male and female trait expression, that is, a range of trait values over which male and female fecundity change in opposing directions with z (e.g., $w_{m}^{\prime }(z)<0$ and $w_{f}^{\prime }(z)>0$ where ′ denotes derivative). In Box 1, we present flexible forms of $w_{m}(z)$ and $w_{f}(z)$ based on power functions that are respectively centered around a male (-θ) and a female (θ) optimum. We will use these functions for our analyses where necessary, in particular for individual-based simulations (we also consider Gaussian functions in Supplementary Appendix, where we obtain equivalent results to those presented in the main text).

### Genetic architecture, genotype-phenotype map, and evolution

We investigate the evolution of trait z under two models of its genetic basis: Kimura’s continuum-of-alleles (Kimura, 1965) model and a polygenic model. We assume initially that all loci are autosomal (non-recombining and recombining segments of sex-chromosomes are considered later in Section “Sex chromosomes and PAR: cold and hotspots for diversifying selection”). These two models, which we detail below, provide complementary insights into the evolution of antagonistic traits by relaxing different assumptions about the genetic architecture of z that are typical of population genetic models of balancing selection (Albert & Otto, 2005; Arnqvist et al., 2014; Connallon & Clark, 2012; Flintham et al., 2021; Fry, 2010; Gavrilets & Rice, 2006; Hill et al., 2019; Hitchcock & Gardner, 2020; Jaquiéry et al., 2013; Jordan & Connallon, 2014; Mullon et al., 2012; Kidwell et al., 1977; Owen, 1953; Otto, 2019; Patten & Haig, 2009; Patten et al., 2010; Rice, 1984; Ruzicka & Connallon, 2020; Spencer & Priest, 2016; Ubeda et al., 2011). Specifically, by allowing a wide range of possible alleles to arise through mutation, our first approach relaxes the assumption that only two alleles with fixed fitness effects may segregate at a sexually antagonistic locus. This enables us to characterize the nature of selection that arises from the male- and female-specific fitness landscapes. Meanwhile, our second model relaxes the assumption that traits are encoded by one or two loci by letting z be polygenic. Doing so allows us to investigate how polygenic variation responds to sex-specific selection.

#### Continuum-of-alleles model

The continuum-of-alleles model (Kimura, 1965) considers z to be encoded by a single additive locus and follows its evolution through recurrent mutations that each create a new allele whose value deviates from the original allele by a random amount (Supplementary Appendix A for details). When mutations are rare, phenotypic evolution under this model can be characterized from an invasion analysis, the basis of which is the geometric growth rate $W(x_{∙},x)$ of a new allelic mutant with phenotypic effect $x_{∙}$ arising in a population otherwise monomorphic for allelic effect x (e.g., Chapter 12 in Otto and Day 2011 for textbook). We assume throughout that allelic effects on phenotype are the same in males and females so that homozygous resident males and females express z=2x while heterozygous mutant males and females express $z_{∙}=x_{∙}+x$. In this case, invasion fitness equals


$$\begin{array}{c} W(x_{∙},x)=\frac{1}{2}\frac{w_{m}(x_{∙}+x)}{w_{m}(2x)}+\frac{1}{2}\frac{w_{f}(x_{∙}+x)}{w_{f}(2x)}, \end{array}$$ (1)

(Supplementary Appendix A Equations (A-1)–(A-5) for derivation, see also Equation (1) in Van Dooren et al., 2004).

The assumption that allelic effects are the same across the sexes generates the strongest possible genetic trade-off between male and female fitness and so provides the most favorable conditions for sexually antagonistic polymorphism. Without this constraint, sexual dimorphism would readily evolve, resolving genetic conflict through the fixation of alleles with sex-specific effects (Connallon & Clark, 2014a; Lande, 1980; Muralidhar & Coop, 2024).

When mutations have small phenotypic effects (such that $x_{∙}$ and x are similar), evolution proceeds in two phases under the continuum-of-alleles model (Dercole & Rinaldi, 2008; Metz et al., 1996; Otto & Day, 2011). Initially, the population evolves under directional selection, whereby positively selected mutations rapidly sweep to fixation so that the population transitions from being largely monomorphic for one trait value to being monomorphic for another. The population may thus converge to a singular trait value $z^{*}=2x^{*}$, which is such that $s(x^{*})=0$ and $s^{\prime }(x^{*})<0$, where $s(x)=\partial W(x_{∙},x)∕\partial x_{∙}{|}_{x_{∙}=x}$ is the selection gradient acting on allelic value (Christiansen, 1991; Eshel, 1983; Geritz et al., 1998; Geritz & Kisdi, 2000). Once the population is fixed for $x^{*}$, it experiences either: (i) stabilizing selection when $h(x^{*})=\partial^{2}W(x_{∙},x^{*})∕\partial {x_{∙}}^{2}{|}_{x_{∙}=x^{*}}\leq 0$, such that the distribution of allelic effects remains unimodally distributed around $x^{*}$; or (ii) disruptive selection, exhibiting negative frequency dependence, when $h(x^{*})>0$ and therefore becomes polymorphic in a process referred to as “evolutionary branching” (Dercole & Rinaldi, 2008; Geritz et al., 1998; Rueffler et al., 2006). In this latter case, selection maintains two alleles that initially encode weakly differentiated phenotypes around $x^{*}$, which then become increasingly divergent as the two alleles accumulate further mutations (Geritz & Kisdi, 2000). We will refer to such negative frequency-dependent disruptive selection as “diversifying selection” (Rueffler et al., 2006) for short.

#### Polygenic diallelic model

In the polygenic model, we assume z is encoded by L loci such that the phenotype expressed by an individual of either sex is given by


$$\begin{array}{c} z=\overset{L}{\underset{k=1}{\sum }}(x_{k,1}+x_{k,2}), \end{array}$$ (2)

where $x_{k,1}$ and $x_{k,2}$ are the allelic values carried at the paternal and maternal copy at locus k, respectively (as in the continuum-of-alleles model, we assume here that allelic effects are fully sex-concordant). Genetic effects on phenotype are thus additive within and between loci, but they may translate into epistatic and dominance effects on fitness, depending on the fecundity functions ($w_{f}(z)$ and $w_{m}(z)$). We assume that each locus k∈{1,…,L} has two possible alleles, ${\text{A}}_{k}$ and ${\text{a}}_{k}$. Allele ${\text{A}}_{k}$ increases the size of the trait z and allele ${\text{a}}_{k}$ decreases it. Specifically, the effect of the allelic copy $x_{k,l}$ inherited from parent l∈{1,2} at locus k is


$$\begin{array}{c} x_{k,l}=\{\begin{array}{cc} \delta_{k}, & \text{if allele }{\text{A}}_{k}\text{ is present}\\ -\delta_{k}, & \text{if allele }{\text{a}}_{k}\text{ is present} \end{array}, \end{array}$$ (3)

where $\delta_{k}>0$ is the absolute phenotypic contribution of a single allele at the locus. We initially assume that each locus contributes equally, with $\delta_{k}=\delta$ for all k, so that the maximum and minimum phenotypes are 2δL and -2δL, respectively, which we assume correspond to the male and female optima, that is, θ=2δL (Supplementary Appendix B for analysis).

With L=1, this model reduces to classic single-locus population genetic models (e.g., Connallon & Clark, 2012; Fry, 2010; Kidwell et al., 1977, see Supplementary Appendix B.1), and with many loci of small effect (L→∞ and δ→0) to quantitative genetic models of sexual antagonism (e.g., Lande, 1980 in the absence of environmental effects on phenotype and with a cross-sex genetic correlation equal to one). Mutations from $A_{k}$ to $a_{k}$ and from $a_{k}$ to $A_{k}$ occur at a per-locus rate of μ. We assume that the L loci are evenly distributed across a single large chromosome, and in the main text that recombination is free between adjacent loci (i.e., occurs at a rate r=0.5). We relax some of these genetic assumptions in Supplementary Appendix C, where we allow for loci to have variable effect sizes (i.e., variation in $\delta_{k}$ across k to model the presence of large and small effect loci, Supplementary Appendix C.1) and for genetic linkage (i.e., r<0.5, Supplementary Appendix C.2). We find that none of these departures from our baseline model have a significant influence on the results presented below (as detailed in the “Discussion” section).

## Results

### Strong sexual antagonism is required for diversifying selection

**Fig 2:**
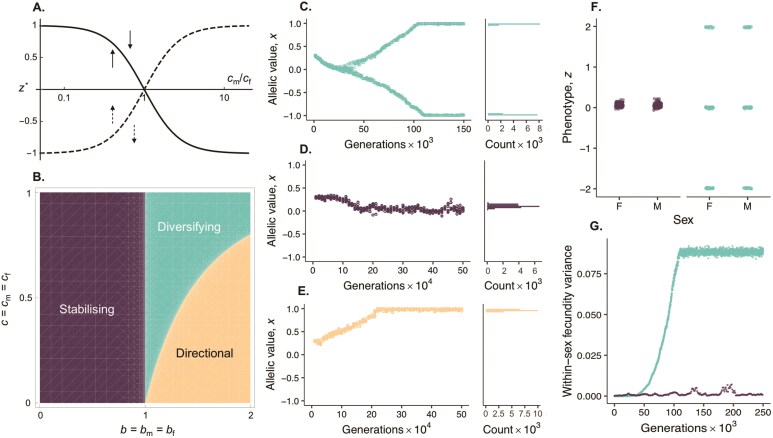
Genetic evolution in a continuum-of-alleles model of sexual antagonism. Panels show mathematical and simulation results for the continuum-of-alleles model when male and female fecundity follow power functions (Equation I.A, Box 1). Panel (A) shows the singular trait value $z^{*}=\theta *\left(1-{\left(c_{m}∕c_{f}\right)}^{\left(1∕1-b\right)}\right)∕\left(1+{\left(c_{m}∕c_{f}\right)}^{\left(1∕1-b\right)}\right)+O(\epsilon^{2})$ (where ϵ>0 is a parameter of the order $c_{m}$ and $c_{f}$) as a function of the fecundity costs in males versus females $c_{m}∕c_{f}$. Solid curve shows $z^{*}$ when it is an attractor of evolutionary dynamics (leading to “diversifying” or “stabilizing” selection, see, for example, Figure 2C and D), and dashed curve shows $z^{*}$ when it is a repellor so that selection favors evolution toward either the female or the male optimum depending on initial condition (“directional selection” see, for example, Figure 2E). Panel (B) shows the parameter space leading to each mode of selection (Supplementary Appendix A for full analysis). Panels (C)–(E) show evolution of allelic values x in a simulation of our continuum-of-alleles model when sexual antagonism leads to diversifying (c=0.9,b=1.5), stabilizing (c=0.05,b=0.5), and directional (c=0.05,b=1.5) selection, respectively. In the left-hand frame of each panel, dots represent a random sample of 10 alleles from the population at intervals of $10^{3}$ (in C) or $10^{4}$ (in D–E) generations. Right-hand frames show the distribution of all alleles in the final generation. Simulations were initialized with all individuals homozygous for an allelic value of x=0.3, with θ=2, Supplementary Appendix A.4.3 for full simulation procedure. Panel (F) shows the phenotypes of a random sample of 50 male and 50 female individuals from a simulation after $2.5\times 10^{5}$ generations under stabilizing (purple dots) and diversifying selection (green dots). Phenotypic variance is much greater under diversifying selection where three types coexist: one expressing the male and another the female optimum, and a third intermediate. Panel (G) shows the average fecundity variance ($Var[w_{m}(z)]∕2+Var[w_{f}(z)]∕2$) through time under diversifying (green dots) and stabilizing (purple dots) selection. The variance in fitness increases when polymorphism arises under diversifying selection. Unless otherwise stated, in all panels, the strength of sex-specific selection is equal across males and females, $c=c_{m}=c_{f}$, and fecundity landscapes are the same shape, $b=b_{m}=b_{f}$.

We first investigate evolutionary dynamics under the continuum-of-alleles model (“Continuum-of-alleles model” section) with arbitrary sex-specific fitness landscapes (i.e., arbitrary $w_{m}(z)$ and $w_{f}(z)$). We characterize the criteria for male and female landscapes to produce diversifying selection and so generate polymorphism in Supplementary Appendices A.2 and A.3. We find that two conditions must be satisfied. First, the population should evolve such that on average individuals express an intermediate trait value $z^{*}=2x^{*}$ for which


$$\begin{array}{c} \frac{w_{f}^{\prime }(z^{*})}{w_{f}(z^{*})}=-\frac{w_{m}^{\prime }(z^{*})}{w_{m}(z^{*})}, \end{array}$$ (4a)

where $w_{u}^{\prime }(z^{*})∕w_{u}(z^{*})$ is the slope of fitness landscape in sex u (Lande & Arnold, 1983). Equation (5a) tells us that at $z^{*}$, a change in trait value has opposing and balanced additive fitness effects across the sexes. For example, under symmetric power functions (Equation I.A in Box 1 with $b=b_{m}=b_{f}$), $z^{*}$ is halfway between the male and female optima when fitness declines with the same intensity away from each sex’s optimum ($c_{m}=c_{f}$); otherwise, $z^{*}$ tends to be closer to the optimum of one sex (Figure 2A).

The second condition for diversifying selection is more restrictive: that $w_{m}(z)$ and $w_{f}(z)$ must have special forms close to $z^{*}$ such that


$$\begin{array}{c} 0<\underset{\text{average fitness curvature}}{\underbrace{\frac{w_{f}^{\prime \prime }(z^{*})}{w_{f}(z^{*})}+\frac{w_{m}^{\prime \prime }(z^{*})}{w_{m}(z^{*})}}}<\underset{\text{square of directional selection strength}}{\underbrace{(\frac{w_{f}^{\prime }(z^{*})}{w_{f}(z^{*})}{)}^{2}+(\frac{w_{m}^{\prime }(z^{*})}{w_{m}(z^{*})}{)}^{2}}}, \end{array}$$ (4b)

where $w_{u}^{\prime \prime }(z^{*})∕w_{u}(z^{*})$ is the curvature of fitness in sex u (Lande & Arnold, 1983). When $w_{u}^{\prime \prime }(z^{*})∕w_{u}(z^{*})>0$, fitness in sex u is accelerating (i.e., convex), whereas it is diminishing (i.e., concave) when $w_{u}^{\prime \prime }(z^{*})∕w_{u}(z^{*})<0$. The middle term of condition (4b) is therefore the curvature in the sex-averaged fitness landscape and indicates whether a population expressing $z^{*}$ is at a local minimum (curvature is positive) or a local maximum (curvature is negative) of this landscape. The right-hand term, meanwhile, is the strength of directional selection in each sex raised to the power of two. Condition (4b) thus tells us that, for selection to be diversifying, fitness must be on average accelerating around $z^{*}$ (middle term) but weakly relative to the strength of directional selection (right-hand term). This reflects the notion that diversifying selection here arises from the tension between two effects: on one hand, disruptive selection across both sexes against intermediate phenotypes (middle term); and on the other, strong competition within each sex that leads to negative frequency-dependent selection on male- or female-adapted phenotypes (right-hand term). Generating the right balance between these forces is difficult, notably because it requires large variation in fitness for both sexes (i.e., large $w_{u}^{\prime }(z^{*})∕w_{u}(z^{*})$). In fact, condition (4b) reveals that whatever the fitness landscape, polymorphism will never arise under the continuum-of-alleles model when directional selection in each sex is weak, that is, where terms of order ${\left[w_{u}^{\prime }(z^{*})∕w_{u}(z^{*})\right]}^{2}$ or higher are negligible, as is often assumed (e.g., Connallon & Clark 2010, 2012; Flintham et al., 2021; Hitchcock & Gardner, 2020; Jordan & Connallon, 2014; Mullon et al., 2012).

In line with these general observations, when sex-specific fitness follows power functions (Equation I.A, Box 1), diversifying selection arises and generates a polymorphism only when fitness is accelerating (b>1) and trades off strongly between males and females ($c_{m}$ and $c_{f}$ large and similar, green regions in Figure 2B and Supplementary Figure 1 top row panels, Figure 2C for an example). Otherwise, selection across the sexes is either stabilizing around $z^{*}$, which occurs when fitness landscapes are diminishing (b<1, Figure 2B, purple region, Figure 2D for an example), or directional toward the male or female optimum, which occurs when landscapes are accelerating (b>1) but selection is not sufficiently strong to maintain polymorphism (Figure 2B yellow region; here $z^{*}$ is a repeller of evolutionary dynamics and z evolves toward θ or -θ depending on the value of z relative to $z^{*}$, Figure 2E for an example). Accordingly, phenotypic and fitness variance within sexes is strongly elevated when sexual antagonism drives diversifying selection but is otherwise low (Figure 2F and G).

### Sexual antagonism diminishes polygenic variation unless selection is diversifying

**Fig 3:**
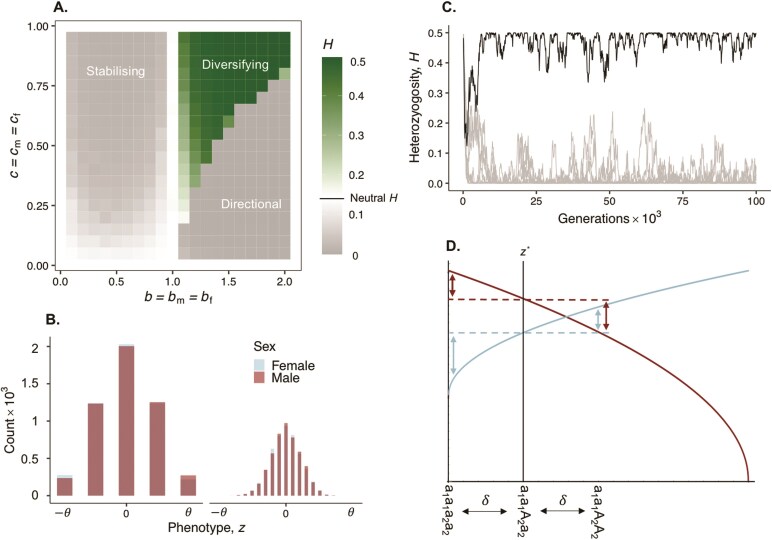
Genetic and phenotypic variation in a polygenic model of sexual antagonism. Panels show simulation results for our polygenic di-allelic model when male and female fecundity follow power functions (Equation (I.A), Box 1). Panel (A) shows average heterozygosity across loci from polygenic simulations. White, green, and gray squares represent average heterozygosity values that are respectively equal, higher, and lower, than that of a neutral locus at mutation-drift balance (Appendix B.3 for full simulation procedures—unless otherwise stated, all panels show results from simulations with L=10 di-allelic loci, δ=1 and $\mu =5\times 10^{-6}$). Panel (B) shows the distribution of male and female phenotypes of a polygenic trait (L=2 on left and L=10 on right) under diversifying selection ($c_{m}=c_{f}=0.9,b=1.5$) after $10^{5}$ generations of a simulation (note that male and female distributions are mostly indistinguishable). Panel (C) shows heterozygosity through time at each individual locus of a polygenic trait (L=10) in a simulation where fitness declines more strongly in males than females ($c_{m}=0.85\times 0.3,c_{f}=0.15\times 0.3,b=0.5$), black curve shows heterozygosity at the “remainder” locus. Panel (D) is a graphic showing male (blue curve) and female (red curve) fecundity under stabilizing selection (b<1) when $c_{m}>c_{f}$ (same parameters as in panel C). Dotted horizontal lines show sex-specific fecundity for individuals expressing the optimal phenotype $z^{*}$ (which here is closer to the male optimum, i.e., $z^{*}<0$). Vertical arrows show the fecundity of double homozygous genotypes in a two-locus model (L=2) compared to intermediate genotypes that are homozygous for the male beneficial allele at locus 2 (${\text{a}}_{2}$) and heterozygous at locus 1. These indicate dominance reversal at locus 2 (with arrows above dashed lines shorter than those below in each sex).

Having characterized the types of selection that can arise from sex-specific fitness landscapes, we next investigate the response when z has a specified genetic architecture, in particular when it is underlain by multiple genes. To do this, we ran individual-based simulations of the polygenic model under a wide range of parameters that modulate genetic conflict (Equation (2) and Equation (3) with Equation (I.A) from Box 1 using SLiM 4 (Haller & Messer, 2023), see Supplementary Appendix B.3 for details and also Supplementary Appendix C.4 for an equivalent analysis with Gaussian fecundity functions). Results of these simulations show that the only conditions under which average heterozygosity across loci is greater than the neutral expectation are those that lead to diversifying selection (i.e., when condition (4b) holds, compare Figure 3A with Figure 2B). Here, heterozygosity is elevated because diversifying selection favors male- and female-adapted genotypes composed of different alleles at many loci, thus maintaining genetic polymorphism across sites. However, because genetic variation is shuffled by Mendelian segregation and recombination at each generation, diversifying selection leads to a wide distribution of phenotypes among individuals that is increasingly smooth and continuous as the number (L) of underlying trait loci increases (Figure 3B). Predictably, the highest levels of heterozygosity and phenotypic variation are observed under conditions where diversifying selection is the most intense, that is, where (i) fitness landscapes are strongly accelerating (b≫1), as this entails that intermediate genotypes show especially low fitness; combined with (ii) exceptionally strong sexual antagonism ($c_{m}closeto1$ and $c_{f}closeto1$) to oppose the fixation of the male or female-adapted genotypes (Figure 3A). In addition, selection is most effective in driving elevated heterozygosity when the number L of loci that contribute to the trait is small relative to population size. This is because otherwise evolution at each individual locus becomes dominated by genetic drift (Supplementary Figure 2).

Diversifying selection that drives high heterozygosity levels also generates a strong sex load (Supplementary Figure 3). For example, a twofold increase in heterozygosity relative to neutrality—equivalent to that produced by a doubling of the mutation rate—is associated with a minimum average fitness reduction of about 20% across the sexes (a significantly stronger load of about 40% is produced when fecundity follows Gaussian functions, dashed vertical line in Supplementary Figure 3).

By contrast, if sexual antagonism does not lead to diversifying selection, average heterozygosity is diminished relative to neutrality. Under directional selection, selection continuously pushes for the optimum of one sex, favoring the fixation of either all A or all a alleles across loci. Meanwhile, stabilizing selection reduces heterozygosity because it favors genotypes that encode the equilibrium phenotype $z^{*}$ in homozygous rather than heterozygous state. This is because homozygotes are more likely to produce offspring also expressing $z^{*}$, that is, their genotypes are less likely to be broken down by random Mendelian segregation (an effect well-known to occur under sex-concordant stabilizing selection Bulmer, 1971; Bürger & Gimelfarb, 1999; Turelli & Barton, 2004). Genetic variation observed in these cases is limited to the ephemeral segregation of alleles owing to recurrent mutation and genetic drift (Supplementary Figure 4A, for example, when L is large or selection weak).

Although stabilizing selection reduces the average heterozygosity across loci relative to neutrality, in some instances, we observe the stable maintenance of a balanced genetic polymorphism at a maximum of one locus (out of the L that code for the trait, Figure 3C). This occurs whenever no homozygous genotype codes for a trait value close enough to $z^{*}$ (Bürger & Gimelfarb, 1999 for a description of this under sex-concordant selection). Polymorphism in this case is maintained at one “remainder” locus that shows sex-specific dominance reversal: both alleles have a larger effect in the sex they benefit than in the sex they harm (owing to the diminishing shape of the fitness landscapes, see Figure 3D for a graphical representation and Supplementary Appendix B.2.2 for analysis of L=2 case). Such a remainder locus reflects a genetic constraint preventing the population from evolving to $z^{*}$. This is the underlying assumption behind population genetics models that see the maintenance of polymorphism at a single sexual antagonistic locus under weak selection (Connallon & Chenoweth, 2019; Fry, 2010; Kidwell et al., 1977). In our polygenic model, however, genetic drift and recurrent mutation drive turnover in the identity of the remainder locus, so that unless selection is particularly strong, any one site shows only transient polymorphism here (Supplementary Figure 4B, also Supplementary Appendix C.1 for this effect when loci vary in their effect size).

Some of the above findings show apparent similarities with those of Turelli and Barton (2004), who analyzed a multilocus population genetics model where selection favors the same optimum in males and females but where alleles can have sex-dependent effects on trait expression, leading them to be potentially sexually antagonistic. It is shown that such sexual antagonism maintains a maximum of one balanced polymorphic locus under weak Gaussian selection and loose linkage (which allows for a “Quasi Linkage Equilibrium approach” Turelli & Barton, 2004). This is the same outcome as seen in our model under weak selection, and the reason for this convergence is that in both cases selection across the sexes is stabilizing when variation in fitness is small (see Supplementary Appendix C.6, Equation (C-27) for the conditions for stabilizing or diversifying selection in Turelli & Barton 2004). However, Turelli and Barton (2004) find no cases of genetic polymorphism when selection is strong (unlike our results under diversifying selection). Specifically, they numerically iterated recursions for 10 additive loci with randomly and independently sampled sex-dependent allelic effects and found that no more than 2 loci ever remained polymorphic in the long run. Presumably, polygenic variation is not maintained in Turelli and Barton (2004) because diversifying selection in their model necessitates large systematic differences in male and female phenotype (see Supplementary Appendix C.6, Equation (C-27)), and parameter combinations leading to such systematic differences are extremely unlikely under the reasonable assumption that allelic effects are independently distributed across loci (as assumed in Turelli and Barton 2004). More broadly, the discrepancy between Turelli & Barton (2004)’s results and ours demonstrates the distinct biology of sexual antagonism that arises from sex-specific landscapes versus sex-specific allelic effects, leading to differences in the scope for balancing selection across loci.

#### Pre-established polymorphisms are unlikely to persist without diversifyingselection

All our analyses thus far assume that the phenotypic distribution in the population is initially unimodal, that is, that individuals rarely differ strongly in their value for z. We relax this in Supplementary Appendix C.5 to consider the consequences of sexually antagonistic selection for two already-differentiated genetic morphs, such as diverged alleles that have arisen through large-effect mutation or gene flow. We show in this Appendix that there exist sex-specific fitness landscapes $w_{m}(z)$ and $w_{f}(z)$ that are such that they do not lead to diversifying selection (i.e., condition (4b) does not hold), but do allow for a pre-established polymorphism at one locus to be robust to small effect mutations. In other words, there are situations where sexual antagonism will stably maintain a coalition of two alleles with highly divergent phenotypic effects although these alleles cannot emerge from gradual evolution under the continuum-of-alleles model. Such situations, however, have limited scope to contribute to genetic variation, and this is for three reasons. First, they require sex-specific fitness landscapes that show very particular properties (Supplementary Appendix C.5.1, Equation (C-20)). Second, these sex-specific fitness landscapes favor polymorphisms that are sensitive to fluctuations in allele frequency (e.g., due to genetic drift) or further large effect mutations (Supplementary Appendix C.5.1, Equation (C-23)). Finally and most importantly, these polymorphisms break down under recombination, such that sexually antagonistic selection here cannot maintain elevated polygenic variation when a trait is encoded by multiple smaller effect loci (Supplementary Appendix C.5.2). This reinforces the notion that balanced sexually antagonistic polymorphism requires either diversifying selection or genetic constraints on allelic values, with polygenic variation being extremely difficult to maintain in the latter case.

### Sex chromosomes and PAR: cold and hotspots for diversifying selection

Our results suggest that the scope for sex-specific selection to maintain polymorphism at autosomal loci is narrow. We therefore extend our analyses to consider sex-linked loci, as they may be more amenable to sexually antagonistic polymorphism. In particular, models of single di-allelic sex-linked loci with arbitrary dominance and selection coefficients have suggested that conditions for balancing selection may be especially lenient on sex chromosomes (Gavrilets & Rice, 2006; Jordan & Charlesworth, 2012; Rice, 1984), usually based on arguments of allelic dominance (e.g., that the absence of male dominance for X-linked promotes the invasion of male-beneficial alleles Rice, 1984, although see Fry, 2010; Ruzicka & Connallon, 2020). This has led to much discussion over whether sex-chromosomes or autosomes should be expected to harbor higher levels of sexually antagonistic variation (Charlesworth et al., 2014; Flintham et al., 2021; Gavrilets & Rice, 2006; Jordan & Charlesworth, 2012; Mullon et al., 2012; Otto et al., 2011; Rice, 1984; Ruzicka & Connallon, 2020).

We first investigate the non-recombining portion of the X-chromosome (or Z-chromosome in ZW species) where such loci do not have Y-linked/W-linked homologs contributing to the same trait (hemizygous regions). We derive the relevant conditions for diversifying selection in Supplementary Appendix D.1. The equations describing these conditions are slightly involved (Equations (D-5) and (D-20) in Supplementary Appendix D) and so we refrain from giving them here, but they reveal that the criteria for diversifying selection are typically less permissive at X-linked than autosomal loci (i.e., compared to Equations (4a) and (4b)). Thus, in contrast to some classic single-locus results (e.g., Rice, 1984; Gavrilets & Rice, 2006), we find the X-chromosome is in fact a coldspot for polymorphism underlying sexually antagonistic traits. This is a direct consequence of the sex-biased inheritance of X-chromosomes (Equation (D-20) in Supplementary Appendix D for formal argument): while females pass on half their X-linked genes to their sons and daughters, males pass on X-linked genes only to daughters. This leads to a bias whereby male-beneficial alleles are disproportionately inherited by females, as these genes are over-represented in high-fecundity fathers. Simultaneously, because males only receive X-linked genes from their mother, an equivalent bias arises whereby female-beneficial alleles tend to be more frequently expressed in males than females because these alleles are over-represented in high fecundity mothers. Sexually antagonistic alleles therefore become associated with the context in which they confer low fitness (i.e., male-beneficial alleles with female carriage and female-beneficial alleles with male carriage), making it more difficult to maintain polymorphism in the population (Avila & Mullon, 2023).

We also consider loci that sit in the “pseudo-autosomal region” (PAR) (Charlesworth et al., 2014; Jordan & Charlesworth, 2012; Otto et al., 2011) of the sex chromosome, linked to a sex-determining region (SDR) with recombination rate r≥0 (Supplementary Appendix D.2 for derivation and Equations (D-22)–(D-23) for conditions for diversifying selection). In short, we find that diversifying selection owing to sexual antagonism occurs more readily on the PAR than on autosomes or on nonhomologous (hemizygous) sex chromosome regions, especially where recombination with the SDR is rare, supporting the notion that these regions are conducive to balancing selection (Charlesworth et al., 2014; Jordan & Charlesworth, 2012). Sexually antagonistic polymorphism in the PAR may even arise under weak selection when linkage with the SDR is tight compared to selection (specifically where r is of the order of the strength of directional selection raised to the power of two, Equations (D-22) and (D-23) in Supplementary Appendix D for details). This greater scope for polymorphism on the PAR is because linkage with an SDR allows male-beneficial alleles to become associated with Y-carriage (and so with male expression), and female-beneficial alleles to become associated with X-carriage (and so, on average, with female expression). This generates a positive correlation between alleles and the genomic context in which they confer high fitness (in contrast to the non-recombining X-chromosome), relaxing the conditions for both alleles to co-exist adaptively in the population (Avila & Mullon, 2023). Nevertheless, while the PAR may be a hotspot for diversifying selection, it is difficult to see how this would translate into the maintenance of polygenic variation, due to the requirement that large numbers of loci affecting the same trait are all in tight linkage with the SDR.

### Sexual antagonism produces weak genomic signatures

Finally, as a last step, we ask whether sexual antagonism at quantitative trait loci that happen to segregate, whether due to mutation-selection-drift balance or to the rare cases of balancing selection, produces signatures that may be found in sequence data. To answer this, we analyzed the simulated data from our polygenic model with two population genomic approaches that are commonly leveraged to identify sexually antagonistic loci, focusing on autosomal loci for simplicity (Supplementary Appendix E for full procedures and calculations). First, we tested whether sexually antagonistic selection drives allelic differentiation between the sexes. This is typically quantified as between-sex $F_{ST}$ (Cheng & Kirkpatrick, 2016; Kasimatis et al., 2019; Lucotte et al., 2016; Mank, 2017a; Ruzicka et al., 2020, 2022), which in the context of sexually antagonistic fecundity selection is measured among the successful parents in a given generation (Ruzicka et al., 2022). Second, we tested whether sexually antagonistic selection leads to detectable associations between allelic state- and sex-specific fitness, as in Genome-Wide Association Studies (GWAS) for sex-specific fitness and sexual antagonism (Berger et al., 2014; Ruzicka et al., 2019, 2020) (our model represents a best-case scenario for this test, as we assume z is under pure genetic control with full heritability). To quantify sex-differences in fitness associations, we computed $\left|\beta_{m,k}-\beta_{f,k}\right|$ for every locus k where $\beta_{m,k}$ and $\beta_{f,k}$ are the linear regression slopes of reproductive success (i.e., number of recruited offspring) in males and females, respectively, against within-individual frequency of allele A at locus k. See Supplementary Appendix E for details on these analyses.

**Fig 4:**
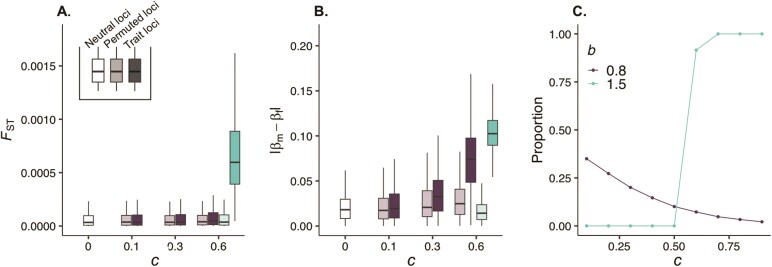
Patterns of allelic variation and fitness associations in polygenic simulations. Panels show results from pooled data for simulations of our di-allelic polygenic model when male and female fecundity follow power functions (Equation (I.A), Box 1, see Supplementary Appendix E for full simulation procedures and calculations, parameters used: $L=10,\delta =1,\mu =5\times 10^{-6}$). Panel (A) shows distributions of allelic differentiation (between-sex $F_{ST}$). Panel (B) shows distributions of absolute sex differences in the linear regression coefficients of fitness against within-individual allele frequency ($\left|\beta_{m,k}-\beta_{f,k}\right|$). In both panels (A) and (B), white boxplots show values for neutral alleles, colored boxplots show values for sexually antagonistic trait loci (purple for b=0.8, green for b=1.5, only parameter values leading to either stabilizing or diversifying selection were used, with diversifying selection arising when fecundity costs were strong enough, c=0.6, to satisfy , see Equation (A-19)), and translucent colored boxplots show values for permuted loci as controls (i.e., trait loci where the sex of each individual is randomly assigned). Boxes show the 25th, 50th, and 75th quantiles; vertical black lines show the interquartile range. Panel (C) shows the proportion of alleles with minor-allele-frequency greater than 0.05 for loci encoding a trait under sexually antagonistic selection.

Figure 4A shows that between-sex allelic differentiation is generally weak for quantitative trait loci under sexually antagonistic selection and that the distribution of $F_{ST}$ across loci is in fact largely similar to that under neutrality. Moreover, this differentiation is also similar to that where either male or female fecundity ($w_{m}$ or $w_{f}$) is randomly assigned for each individual regardless of their sex. This is expected, as generating appreciable between-sex $F_{ST}$ values typically requires strong sex-specific selection acting on a given locus (Kasimatis et al., 2017, 2019), and sexually antagonistic selection acting on a polygenic trait translates into weak effects on each individual locus. One exception is where sexual antagonism causes strong diversifying selection (Figure 4A, green boxplots). This leads to elevated allelic differentiation among the sexes, although this effect declines significantly with the number of loci encoding a trait (e.g., with $c_{m}=c_{f}=0.6$ an $F_{ST}$ of the order of $10^{-2}$ is generated at a single locus, see Figure 5 in Kasimatis et al., 2019, but only an $F_{ST}$ of the order $10^{-4}$ when L=10, Figure 4A here). This indicates that measures of genetic differentiation such as sex-specific $F_{ST}$ will produce a signal for sexually antagonistic quantitative trait loci only under the relatively extreme biological scenarios that lead to diversifying selection. However, because polygenicity spreads selection thinly across loci, this signal is nonetheless weak, and detecting it will require the availability of particularly large datasets or methods that combine $F_{ST}$ patterns across the genome (e.g., by relating to the degree of biased gene expression Cheng & Kirkpatrick, 2016).

When feasible, performing genetic associations with fitness may offer a more productive approach. Indeed, we observe that compared to controls, sexual antagonism drives an increase in $\left|\beta_{m,k}-\beta_{f,k}\right|$ when selection is either stabilizing or diversifying, so long as selection is sufficiently strong (i.e. $c_{m}$ and $c_{f}$ not too small, Figure 4B). Sex-specific fitness associations are possible when selection is stabilizing for $z^{*}$ because fitness landscapes in each sex can be steep close to $z^{*}$ (e.g., see Figure 1 Top where $z^{*}=0$). As a consequence, genetic variation segregating either at a remainder locus or ephemerally owing to mutation and drift can covary noticeably with male and female fitness in opposite ways. However, detecting sexually antagonistic loci when stabilizing selection is strong may prove challenging. This is because, outside of a remainder locus, selection is effective in eliminating variation in this case, such that most loci either fix or show low minor allele frequency (Figure 4C), leaving them unamenable to statistical calculations unless large volumes of data are available. Together, our results thus support the notion that genomic approaches for identifying sexually antagonistic alleles show strong limitations (Kasimatis et al., 2019, 2021; Ruzicka et al., 2020, 2022). In particular, they highlight that, owing to a lack of balancing selection, detecting antagonistic loci underlying polygenic traits is likely to be even more difficult than suggested by prior single-locus theory (Ruzicka et al., 2020 for review).

## Discussion

### A narrow scope for sexual antagonism to maintain genetic variation

Our analyses indicate that sex-specific selection on shared continuous traits can drive above-neutral levels of genetic variation, but that this occurs only under restrictive conditions that should not commonly be met in natural populations. This is because diversifying selection is needed to maintain elevated heterozygosity when traits depend on many alleles and such selection typically entails strong conflict over trait expression between males and females. That is, selection in each sex must act in opposing directions, be both strong and of similar intensity in both sexes and must occur on an accelerating fitness landscape: a restrictive combination of conditions. For example, using datasets of collated sex-specific selection gradients from wild populations (634 trait estimates Singh & Punzalan, 2018) and humans (34 trait estimates Sanjak et al., 2018), we found no traits for which estimated selection gradients were consistent with diversifying selection (i.e., we found no traits for which selection gradients satisfied , Supplementary Appendix F for details).

Moreover, where sexual antagonism is strong enough to generate diversifying selection, it should rarely last over substantial evolutionary periods. This is because random Mendelian segregation leads phenotypic variation in a polymorphic population to remain distributed around an intermediate value (Figure 3B) that confers especially low fitness in both sexes. Consequently, even a modest boost to heterozygosity in a polygenic sexually antagonistic trait is associated with a strong depression in mean fitness (Supplementary Figure 3). Owing to this large load, selection to resolve conflict (Bonduriansky & Chenoweth, 2009; Lande, 1980) (through, e.g., the evolution of sex-specific gene expression Mank, 2017b) is very strong. Therefore, such polymorphism is unlikely to persist over extended evolutionary periods unless there are extreme constraints on genetic architecture that preclude sexual dimorphism (Lande, 1980; Muralidhar & Coop, 2024).

If conflict over trait expression is not strong enough to elevate heterozygosity in a continuous trait, sexual antagonism acts to erode genetic variation. When this happens, balanced sexually antagonistic polymorphism can be maintained only in two specific cases. First, when sexual antagonism causes stabilizing selection for an optimal phenotype that is unattainable by any homozygote, polymorphism can develop at a maximum of one remainder locus owing to patterns of allelic dominance (sex-specific dominance reversal for an autosomal locus, Figure 3C and D, and recessivity in females for an X-linked locus, Supplementary Appendix D; see Connallon and Clark 2014b; Fry 2010; Kidwell et al. 1977; and Rice 1984 for single locus models). However, variation is unlikely to be long-lasting at any given site as mutation and drift drive regular turnover in the identity of the remainder locus (Supplementary Figure 4, Supplementary Appendix C.1), and because polymorphism is not robust to the appearance of new alleles that encode the optimal phenotype when homozygous. Second, polymorphism can occur at a locus when two highly diverged alleles are present before the onset of sexual antagonism, or arise through large-effect mutations or through gene flow (Supplementary Appendix C.5 for details). However, these allele coalitions are especially sensitive to fluctuations in allele frequency (e.g., due to genetic drift) or to further large-effect mutations, so a polymorphism of this sort is also unlikely to show long-term persistence.

In fact, the only circumstance where we find permissive conditions for elevated sexually antagonistic polymorphism when multiple alleles affect a trait is when loci are in tight linkage with the sex-determining region (for di-allelic models of this, see Charlesworth et al., 2014; Jordan & Charlesworth, 2012). Diversifying selection can occur here because genetic linkage allows sexual antagonism to drive an association between the sex in which an allele is beneficial and the sex in which an allele resides more commonly so that polymorphism becomes easier to maintain in the population (Avila & Mullon, 2023). In this way, our results echo conditions for the maintenance of polymorphism owing to local adaptation to two different environments connected by dispersal (e.g., Van Doorn & Dieckmann, 2006; Lythgoe, 1997; Meszéna et al., 1997; Spichtig & Kawecki, 2004; Svardal et al., 2015). In these models, polymorphism occurs only under limited dispersal (analogous to the recombination rate being low here) as this allows alleles to become over-represented in the environment in which they are adaptive; otherwise, gene flow breaks this association and thus inhibits polymorphism. (In fact, a two-patch local adaption model with soft selection Débarre & Gandon, 2011 and random dispersal is equivalent to a model of autosomal sexual antagonism Connallon et al., 2018, and with limited dispersal to a model of sex-specific selection on the PAR, see Supplementary Appendix C.6 and G for details and further discussion on this connection.) Nevertheless, we do not expect diversifying selection in the PAR to drive polygenic polymorphism, due to the requirement that multiple loci influencing the same trait be present in this region and in tight linkage with an SDR.

Altogether, our results indicate that not only are the conditions for polygenic variation restrictive (requiring strong sexual antagonism) but also that the long-term maintenance of sexually antagonistic polymorphism at any locus requires strong genetic constraints: (1) Constraints on gene expression that maintain strong intersexual genetic correlations, that is, such that diversifying selection will not quickly drive the evolution of sexual dimorphism; (2) Constraints on the possible allelic variants that may arise at an antagonistic locus (in order for polymorphism at a remainder locus to arise under stabilizing selection); and (3) Constraints on the rate of recombination between an antagonistic locus and the sex-determining region (allowing for tight linkage between these loci and therefore for diversifying selection without imposing a strong sex load). This conclusion is in spite of a number of assumptions in our model that maximize the opportunity for sexual antagonism to produce balancing selection; namely a perfect intersexual genetic correlation and the absence of environmental effects on trait expression. Without either of these assumptions, the scope for strong and enduring sexual antagonism is significantly reduced (Lande, 1980; Muralidhar & Coop, 2024), indicating our results are in fact conservative. Moreover, our conclusions were also robust to relaxing two constraints from our polygenic model (Supplementary Appendix C): that alleles at all loci have equal phenotypic effect sizes and that recombination between loci is free. We found that neither of these assumptions qualitatively affected the parameter conditions for polymorphism (we discuss implications of the ecological and genetic assumptions of our model in depth in Supplementary Appendix G).

Our conclusions may appear at odds with previous results stemming from other models based on fitness landscapes (Connallon & Clark, 2014b; Sellis et al., 2011). In particular, Conallon and Clark (2014b) consider a continuous trait evolving on a two-sex fitness landscape. This trait may be polygenic, but it is assumed that mutations are rare enough that only one site segregates at a time (so that the trait can only ever show polymorphism at a single locus). They calculate the probability that a mutation that arises in a monomorphic background and that may have sex-specific effects on the trait experiences balancing selection. They find that this probability can be high owing to sex-specific dominance reversal for fitness that emerges from concave/saturating landscapes close to the optima (see also Connallon and Chenoweth 2019). These insights have led multiple authors to suggest that sexual antagonism can drive genetic variation in polygenic traits due to balancing selection (e.g., Arnqvist et al., 2014; Connallon & Clark, 2014b; Connallon & Chenoweth, 2019; Grieshop & Arnqvist, 2018; Mank, 2017a; Kaufmann et al., 2023). However, our analyses show that balancing selection here is a direct consequence of the genetic constraints imposed by the assumption that only a single locus ever segregates. Sexual antagonism acting on a polygenic trait in fact typically opposes genetic variation across loci, leading to a balanced polymorphism at a maximum of one locus irrespective of the number of loci encoding a trait. In other words, while concave/saturating fitness landscapes may be conducive to single-locus polymorphisms, they do not drive elevated genetic variation in a polygenic trait. Rather, selection must be diversifying, which entails very different landscapes to those highlighted in previous models: requiring accelerating/convex and steep fitness curves. However, because of the need for strong selection and the size of the ensuing sex load, we expect that these landscapes will be rare and that antagonisms arising on them will be quickly resolved.

In the absence of balancing selection, single-locus population genetics models have also been used to argue that genetic variation can persist more easily at mutation-selection-drift balance when selection is sexually antagonistic versus sexually concordant or sex-limited (Connallon & Clark, 2012; Mullon et al., 2012). Our results show that this is true over short evolutionary timescales when a population is under directional selection (i.e., when it is displaced from the optimal phenotype $z^{*}$). This is because directional selection at a locus (i.e., favoring the fixation of one allele over the other) is—all else equal—weaker when this selection arises from antagonism rather than concordance as directional selection within each sex is acting in different directions. In the context of our model, this can be seen from the selection gradient on a trait experiencing sex-specific selection (Equation (A-10) in Supplementary Appendix A.2), which indicates that directional selection is weaker under sexually antagonistic than sexually concordant or sex-limited selection, leading to slower convergence of a population to an optimal phenotype $z^{*}$ and higher levels of standing variation at mutation-selection-drift balance during this phase (see Supplementary Appendix A.2 for details). However, this intuition does not hold in the long term once a population experiences stabilizing selection around $z^{*}$. This is because the strength of stabilizing selection for $z^{*}$ depends only on the curvature of the male and female fitness landscapes (Equation (A-13) in Supplementary Appendix A.2) and thus is independent of the direction of selection acting in each sex. Consequently, once a population experiences stabilizing selection, there is no reason to expect the levels of standing variation at mutation-selection-drift balance will fundamentally differ depending on whether selection across the sexes is antagonistic or concordant (all else being equal).

### Empirical implications

Our analyses provide several insights for empirical work on sexually antagonistic polymorphism. First, our results indicate that traits under sexually antagonistic selection will typically exhibit relatively little additive genetic variation. Where sexually antagonistic traits are found to show high levels of standing variation at autosomal loci, we expect such traits to be under strong selection with fitness landscapes being on average accelerating across the sexes, two necessary conditions for generating diversifying selection. Empirically, this could be tested directly by fitting quadratic regressions of reproductive success on trait values in each sex (Lande & Arnold, 1983). We note, however, that nonlinear selection gradients on sexually antagonistic traits are rarely significant (e.g., Lewis et al., 2011; Stearns et al., 2012; Tarka et al., 2014), likely due to the requirement of large datasets to detect second-order fitness effects. In principle, the effect of sexual antagonism on levels of genetic variation could also be assessed experimentally, by measuring the impact of manipulating the strength and nature of antagonistic selection on levels of additive genetic variation for fitness. Studies using family-based selection for sexual dimorphism (e.g., Kaufmann et al., 2021, 2023) provide templates for such an approach. One such study (Kaufmann et al., 2023) found that standing genetic variation did decrease under antagonistic selection, albeit less so than under other modes of selection.

At the genetic level, our results suggest that antagonistic selection on continuous traits leaves few signatures in patterns of polymorphism within populations—except in the presence of very strong diversifying selection (Figure 4). Therefore, in most cases, genetic signatures of sexual antagonism will be subtle and correspond mostly to allelic variation that is maintained due to mutation. Whether and to what extent these signals are detectable will depend on the circumstances. Most importantly, detectability will depend on a number of experimental parameters that have predictable effects on statistical power and that we have not explored here, such as sample size and the degree of environmental noise in the trait. Beyond these, our results indicate that the approach used to detect genetic signatures will have a major impact on power. In particular, we find that both methods based on between-sex genetic differentiation (e.g., sex-specific $F_{ST}$  Cheng & Kirkpatrick, 2016; Lucotte et al., 2016; Mank, 2017a; Kasimatis et al., 2019; Ruzicka et al., 2020, 2022) and fitness associations (e.g., GWAS for sex-specific fitness Berger et al., 2014; Ruzicka et al., 2019, 2020; Sanjak et al., 2018; Zhu et al., 2023) are constrained by the fact that sexual antagonism typically reduces genetic variation and so leaves few loci at which any detectable signal of sexual conflict would be present. Indeed, these signals may easily be drowned in genomic background noise unless a candidate set of loci is known. Even where a locus is detected, it would necessarily provide a poor picture of the loci that are subject to antagonistic selection, which will include many lacking variation. Furthermore, when sexually antagonistic variation does segregate (either due to mutation or balancing selection), sex-specific $F_{ST}$ methods likely have especially low power to detect individual trait loci that contribute to phenotypes with a wide genetic basis. This is because such a signal is based on frequency change over a single generation (Kasimatis et al., 2019) and selection on individual loci underlying a polygenic trait is weak unless sexual antagonism leads to extremely strong diversifying selection at the phenotypic level (although this may be less problematic for approaches looking at genome-wide signs of sex-specific selection and so gain power across loci Cheng & Kirkpatrick, 2016). Meanwhile, approaches based on fitness associations (e.g., GWAS for sex-specific fitness Berger et al., 2014; Ruzicka et al., 2019; Sanjak et al., 2018; Ruzicka et al., 2020; Zhu et al., 2023) are more powerful—relatively speaking, but nonetheless, the small per-locus effects of sexual antagonism also compromises their usefulness where selection is not strong.

As a corollary to our finding that sexually antagonistic selection acting on a given trait maintains at most a single polymorphic locus (unless selection is strong and diversifying, Figure 4C), species exhibiting multiple sexually antagonistic loci with elevated levels of genetic variation likely do so because they experience antagonistic selection on several independent traits. This is consistent with antagonistic loci detectable by GWAS approaches showing no functional enrichment (Ruzicka et al., 2019). In addition, the predicted turnover in the identity of trait loci harboring polymorphism at any given time under stabilizing selection fits with the observation that polygenic variation underlying traits under antagonistic selection shows little signal of long-term balancing selection (Ruzicka et al., 2022; Sylvestre et al., 2023). Our results, therefore, suggest that the rare loci where a strong signal of antagonism and balancing selection is detectable are expected to be those showing large phenotypic (and hence fitness) effects. It is these loci that are most likely to generate the genetic constraints that are necessary to maintain long-term polymorphism under stabilizing selection across the sexes. Indeed this is true for allelic variants at the *VGLL3* gene in Atlantic salmon that generate a sexually antagonistic polymorphism in maturation time and account for 39% of trait variation (Barson et al., 2015). Similarly, large and experimentally replicable effects were demonstrated at the *DsFAR2-B* locus that underlies a sexually antagonistic polymorphism in cuticular hydrocarbon profiles in the tropical fly *Drosophila serrata* and that shows signatures of balancing selection (Rusuwa et al., 2022).

## Conclusions

Previous theory on sexual antagonism has relied overwhelmingly on models of Mendelian traits that are determined by a limited number of alleles with fixed effects (Albert & Otto, 2005; Arnqvist et al., 2014; Connallon & Clark, 2012; Flintham et al., 2021; Fry, 2010; Gavrilets & Rice, 2006; Hill et al., 2019; Hitchcock & Gardner, 2020; Jaquiéry et al., 2013; Jordan & Connallon, 2014; Kidwell et al., 1977; Mullon et al., 2012; Otto, 2019; Owen, 1953; Patten & Haig, 2009; Patten et al., 2010; Rice, 1984; Ruzicka & Connallon, 2020; Spencer & Priest, 2016; Ubeda et al., 2011). This reliance may have led to the expectation that sexually antagonistic genetic polymorphisms are a regular feature of sexual species (Arnqvist et al., 2014; Connallon & Clark, 2014b; Connallon & Chenoweth, 2019; Fry, 2010; Grieshop & Arnqvist, 2018; Grieshop et al., 2024; Kaufmann et al., 2023; Rice, 1984) and that such polymorphisms should be detectable from genomic data (Mank, 2017a; Rowe et al., 2018; Ruzicka et al., 2020). Our work has shown that this expectation rarely extends to continuous traits. By considering some of the complexity behind such traits, our model lays bare that previous conditions for polymorphism are direct consequences of constraining the number and the nature of alleles that can affect a trait. These conditions do not generalize to cases where a wider variety of alleles can arise either within or across loci. In fact, our analyses indicate that sexual antagonism is not a straightforward driver of the long-term maintenance of polymorphism at loci underlying continuous traits and rarely produces sex-specific patterns of variation outside of sex-determining regions. We, therefore, suggest that most sexually antagonistic polymorphisms associated with quantitative trait loci are transient and should be observed no more frequently than at neutral sites, except when males and females face strongly divergent fitness requirements and impregnable genetic constraints are operating.

## Supplementary material

Supplementary material is available online at *Evolution Letters*.

## Supplementary Material

qrae059_suppl_Supplementary_Material

## Data Availability

No new data were generated or analyzed in support of this research. An example SLiM simulation script and an R script used for analyses in Supplementary Appendix F are included as supplementary files.
